# Extending the Developmental Origins of Health and Disease theory: does paternal diet contribute to breast cancer risk in daughters?

**DOI:** 10.1186/s13058-016-0760-y

**Published:** 2016-10-12

**Authors:** Stephanie Romanus, Patrick Neven, Adelheid Soubry

**Affiliations:** 1 Epidemiology Research Group, Department of Public Health and Primary Care, Faculty of Medicine, KU Leuven - University of Leuven, 3000 Leuven, Belgium; 2 Division of Gynaecological Oncology, Leuven Cancer Institute, KU Leuven - University of Leuven, Leuven, Belgium

**Keywords:** DOHaD, Epigenetics, Paternal influences

## Abstract

The Developmental Origins of Health and Disease (DOHaD) theory focuses on the consequences of periconceptional and in utero exposures. A wide range of environmental conditions during early development are now being investigated as a driving force for epigenetic disruptions that enhance disease risk in later life, including cardiovascular, metabolic, endocrine, and mental disorders and even breast cancer. Most studies involve mother–child dyads, with less focus on environmental influences through the father. Over the last few years, however, new insights have been introduced on paternal effects and the plasticity of the epigenome of developing sperm cells have been proposed to underlie inheritable changes from ancestral exposures. The field is evolving rapidly and study results from animal models are promising. Although caution should be taken in translating animal data to humans, epidemiological findings also suggest a prominent role of the father. Therefore, we here propose an extension to the DOHaD theory to include also paternally inheritable influences.

## Main text

Inherited transgenerational non-genetic changes may be nature’s response to adapt relatively quickly to environmental variations [1]. However, if an individual is exposed to an adverse environmental factor, such as malnutrition, during a critical developmental stage in life, the risk of developing a chronic disease or cancer in their offspring may increase [2]. Results of a study published in a recent issue of *Breast Cancer Research* by Fontelles et al. [3] contributes to a new area of research on environmentally induced risk for chronic diseases in offspring through the father. Thomas Prates Ong, Sonia de Assis, and associates showed that an animal-based high-fat diet in rodents increases breast cancer risk in daughters after administration of the carcinogen 7,12-dimethylbenz[a]anthracene (DMBA) [3, 4]. Although these new findings suggest paternal influence on breast cancer risk in daughters, care should be taken when interpreting these results. The DMBA carcinogen-induced model is the current mainstay for studies on cancer development, but it is still only an approach to mimic human breast cancer. On the one hand it does not include all features and molecular pathways involved in human tumors and, on the other, animal cancer models may exceed natural human responses and hence induce artifactual effects. Furthermore, time frames of susceptibility to environmental insults are different in humans and rodents. Hence, extrapolations from a single animal strain to humans require further investigation. Other reasons for concern are failure to randomly allocate animals for intervention or analytic procedures, biased selection of outcome measures, and confounding. For instance, outcome measures in Fontelles et al.’s study may be affected by metabolic changes and disturbances in glucose homeostasis, a reason for the authors to look into the insulin-like growth factor-1 (IGF-1) pathway and receptor blockade. Interestingly, Zhu et al. [5] showed that energetics interventions in rat result in a reduction of mammary carcinogenesis. Another reason for concern is the lack of statistical testing for multiple comparisons. It is important to address these disadvantages and substantially improve methodological approaches, especially because animal studies often provide the rationale for clinical and epidemiological studies.

While studies regarding nutritional effects on cancer risk are limited, a number of experiments in animal models strongly indicate that a suboptimal diet during male gametogenesis can influence male fertility, delay embryonic development, and result in offspring with impaired glucose metabolism, increased body weight, or other adverse metabolic outcomes [6–9]. Epigenetic mechanisms are considered as the underlying cause. For instance, when male rats were administered a folate-deficient diet before mating, a decrease in global DNA methylation in the liver of the offspring was detected [10]. The group of Romain Barrès showed that a high-fat diet in male rats changes the epigenome of sperm. Most interestingly, these diet-induced epigenetic responses were paralleled by transgenerational inheritance of metabolic dysfunction throughout two generations [11]. Fullston et al. [12] showed an interesting phenomenon where a “second hit” of high-fat diet consumption by an F1 male that was sired by an obese father exacerbated metabolic and fertility outcomes. This suggests that consumption of an obesogenic diet identical to that of the father does not result in a protective response in the offspring. Instead, predisposition for metabolic and reproductive phenotypes worsened.

Nearly simultaneously, studies on epidemiological data provided evidence that the human paternal genome is also sensitive to environmental influences. Longitudinal studies suggest that fathers and grandfathers affect descendants’ health through their diet or body fat composition; health effects include increased body fat changes in premenarcheal daughters [13] and shorter life span because of diabetes or cardiovascular diseases [14]. These gender-specific associations suggest that also in humans intergenerational associations are related to germ line changes, rather than just socio-economic factors. Paternal obesity has also been determined to be an independent risk factor for autism spectrum disorder in offspring [15]. Although it is not always possible to distinguish maternal and paternal influences, malnutrition around conception has been associated with breast cancer in the Dutch famine cohort [16]. The Newborn Epigenetic STudy (NEST) cohort provided the first evidence that newborns born to obese fathers had altered methylation profiles at imprinted genes important in embryonic growth and cancer development [17, 18]. Recently, two independent studies linked these paternal obesity-related epigenetic differences with altered epigenetic profiles in sperm [19, 20].

The contribution of paternal obesity to human development or disease susceptibility is especially relevant due to the global obesity trend and thus merits further exploration. Given the epigenome is malleable, early intervention procedures could prevent an epigenetically inherited increased susceptibility to cancer or other diseases. For instance, supplementation of methyl donors, including folic acid and vitamin B12, to future mothers to reduce the risk of congenital defects has been a major focus of public health agencies for many years, but the potential effects of paternal deficiencies in folate or other nutrients have not attracted any attention yet. Little is known about the consequences of the paternal diet on the sperm epigenome. Hence, future fathers do not receive any public health recommendations. In order to improve our understanding of paternal epigenetic influences on DOHaD, research strategies should be planned in an interdisciplinary research environment including oncologists, molecular biologists, nutritionists, biostatisticians, and epidemiologists. More studies on different environmental (nutritional) compounds and their effects on specific windows of development, through sperm and early embryo developmental stages, are needed to confirm and better understand current observations. It needs to be noted that parents should not be blamed for the health status of their children, but if dietary manipulations could prevent certain diseases or cancer, it is important to further unravel the epigenetic processes involved in the maternal and paternal contribution of trans- or intergenerational epigenetic inheritance. Hence, the international society for DOHaD would benefit from an addition on preconceptional paternal exposures, besides maternal contributions. Therefore, we suggest an extension of the DOHaD theory.

## Abbreviations

DOHaD: Developmental Origins of Health and Disease
DMBA: 7,12-dimethylbenz[a]anthracene
IGF-1: insulin-like growth factor-1
